# Molecular Basis of Overdominance at a Flower Color Locus

**DOI:** 10.1534/g3.117.300336

**Published:** 2017-10-19

**Authors:** Amy M. LaFountain, Wenjie Chen, Wei Sun, Shilin Chen, Harry A. Frank, Baoqing Ding, Yao-Wu Yuan

**Affiliations:** * Department of Ecology and Evolutionary Biology, University of Connecticut, Storrs, Connecticut 06269; † Department of Chemistry, University of Connecticut, Storrs, Connecticut 06269; ‡ Key Laboratory of Adaptation and Evolution of Plateau Biota, Northwest Institute of Plateau Biology, Chinese Academy of Sciences, Xining 810008, Qinghai, China; § Key Laboratory of Crop Molecular Breeding of Qinghai Province, Xining 810008, Qinghai, China; ** Institute of Chinese Materia Medica, China Academy of Chinese Medical Sciences, Beijing 100700, China

**Keywords:** heterosis, *Mimulus*, *FLAVONE SYNTHASE*, antagonistic pleiotropy, anthocyanins

## Abstract

Single-gene overdominance is one of the major mechanisms proposed to explain heterosis (*i.e.*, hybrid vigor), the phenomenon that hybrid offspring between two inbred lines or varieties show superior phenotypes to both parents. Although sporadic examples of single-gene overdominance have been reported over the decades, the molecular nature of this phenomenon remains poorly understood and it is unclear whether any generalizable principle underlies the various cases. Through bulk segregant analysis, chemical profiling, and transgenic experiments, we show that loss-of-function alleles of the *FLAVONE SYNTHASE* (*FNS*) gene cause overdominance in anthocyanin-based flower color intensity in the monkeyflower species *Mimulus lewisii*. FNS negatively affects flower color intensity by competing with the anthocyanin biosynthetic enzymes for the same substrates, yet positively affects flower color intensity by producing flavones, the colorless copigments required for anthocyanin stabilization, leading to enhanced pigmentation in the heterozyote (*FNS/fns*) relative to both homozygotes (*FNS/FNS* and *fns/fns*). We suggest that this type of antagonistic pleiotropy (*i.e.*, alleles with opposing effects on different components of the phenotypic output) might be a general principle underlying single-gene overdominance.

It has been known for >100 yr that intercrosses between divergent inbred lines or varieties within a species can often yield hybrid offspring with stronger performance than both parents (Darwin 1876), a phenomenon termed heterosis or hybrid vigor (East 1908, 1936; Shull 1908, 1914). Due to its obvious value for crop improvement, heterosis has been utilized extensively in agriculture in the past century (Duvick 1999). Two long-standing hypotheses are routinely discussed in explaining the genetic bases of heterosis (East 1908, 1936; Shull 1911; Jones 1917; Crow 1948; Birchler *et al.* 2003; Lippman and Zamir 2007). The “dominance” hypothesis proposes that deleterious recessive alleles at many loci in each parent are complemented by the dominant alleles from the other parent, resulting in better performance in the hybrid as a whole. In contrast, the “overdominance” model argues that allelic interactions at potentially a single locus could lead to a stronger phenotype in the heterozygote than both homozygous parents. A third model emphasizing the role of epistatic interactions among loci also has gained substantial support (Schnell and Cockerham 1992; Yu *et al.* 1997; Goff and Zhang 2013). Among these hypotheses, single-gene overdominance remains the most enigmatic from a mechanistic viewpoint. However, this model is perhaps the most attractive from an applied perspective, as single-gene manipulations could theoretically lead to the improvement of agricultural crops (Krieger *et al.* 2010; Park *et al.* 2014). Although several convincing examples of single-gene overdominance have been reported over the decades [reviewed in Liberatore *et al.* (2013)], the molecular nature of this phenomenon is far from resolved (Goff and Zhang 2013; Liberatore *et al.* 2013). Perhaps the most widely discussed example of single-gene overdominance is that of human sickle cell anemia. A hemoglobin mutation negatively affects human survival by causing sickle cell anemia, but positively affects human survival by protecting against malaria (Pauling *et al.* 1949; Aidoo *et al.* 2002; Ferreira *et al.* 2011). In this case, human survival is the phenotype of interest; sickle cell anemia and resistance to malaria are two important components that determine the final phenotypic output. The strongest phenotype is achieved in the heterozygote by balancing the antagonistic effects of the mutant allele. This model of antagonistic pleiotropy was first described to explain the maintenance of human senescence (Williams 1957), and later coopted as a mechanism of overdominance to explain the prevalence of some human disease alleles (*e.g.*, Huntington’s disease and cystic fibrosis) (Carter and Nguyen 2011). However, this notion was rarely considered as a potential mechanism underlying the sporadically reported cases of overdominance (Liberatore *et al.* 2013) outside of the context of human disease dynamics.

To gain more mechanistic insights into single-gene overdominance, we have analyzed a flower color locus of the monkeyflower species *Mimulus lewisii*. The *M. lewisii* inbred line LF10 bears light pink flowers due to anthocyanin accumulation in the petal lobes (Figure 1A). Three allelic, ethyl methanesulfonate (EMS) mutants generated in the LF10 background are phenotypically indistinguishable and display a clear overdominant effect in flower color intensity: the homozygous mutant produces very pale flowers, whereas the heterozygote has flower color darker than both the wild-type and the homozygous mutant (Figure 1A).

**Figure 1:**
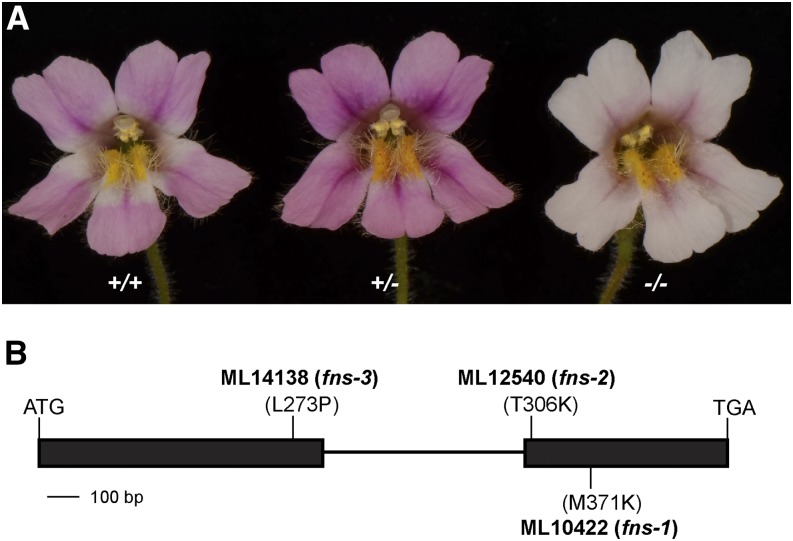
Mutant *fns* alleles of *Mimulus lewisii* cause overdominance in flower color intensity. (A) Flower phenotype of the wild-type LF10 (+/+), the heterozygous mutant (+/−), and the homozygous mutant (−/−). (B) The three allelic mutants (ML10422, ML12540, and ML14138) all harbor nonconservative amino acid replacements at highly conserved sites of *FNS* (also see Figure S1 in File S1).

Floral anthocyanin pigmentation is fairly-well understood in *M. lewisii*. Loss-of-function mutations in the transcriptional activators (*i.e.*, the R2R3-MYB, bHLH, and WD40 regulatory complex) of the anthocyanin biosynthetic pathway (ABP) lead to very pale or white flowers (Yuan *et al.* 2014). Dark pink flowers can be produced by downregulation of the ABP repressor, *ROSE INTENSITY1* (*ROI1*) (Yuan *et al.* 2013a), or alternatively, by knocking down the expression level of *FLAVONOL SYNTHASE* (*FLS*) (Yuan *et al.* 2016), which encodes an enzyme that competes with the anthocyanin biosynthetic enzymes for the same substrate, dihydroflavonol. However, none of these previously characterized genes was known to have an overdominant effect in flower color intensity, indicating a yet to be discovered gene underlying the overdominant phenotype. Here, we report *FLAVONE SYNTHASE* (*FNS*) as the causal gene, and suggest antagonistic pleiotropy as a general mechanism of single-gene overdominance.

## Materials and Methods

### Plant materials

The *M. lewisii* inbred line LF10 (wild-type) was described in Yuan *et al.* (2013a). The three EMS mutants (ML10422, ML12540, and ML14138) were generated using LF10, following Owen and Bradshaw (2011). Plants were grown in the University of Connecticut EEB research greenhouses under natural light supplemented with sodium vapor lamps, ensuring a 16 hr day length.

### Genomic analyses for causal gene identification

Previously, we have used bulk segregant analysis coupled with Illumina sequencing to map the causal genes underlying *M. lewisii* EMS mutants that were generated in the LF10 background (Yuan *et al.* 2013b; Sagawa *et al.* 2016; Ding *et al.* 2017; Stanley *et al.* 2017). All previous bulk segregant analyses were performed on F_2_ populations between a mutant and the mapping line SL9. Unfortunately, SL9 produces pale pink flowers, which may confound phenotype scoring in this case since the focal phenotype of this study involves flower color intensity. To circumvent this problem, we employed a novel, hybrid strategy that combines bulk segregant analysis of an “LF10 × ML10422” F_2_ population and genome comparisons between multiple mutants.

An LF10 × ML10422 F_2_ population (455 individuals) was grown to flower. Flower color segregated as follows: 112 light pink (homozygous for the wild-type allele), 235 dark pink (heterozygous), and 98 very pale (homozygous for the mutant allele). DNA samples from 96 very-pale individuals (*i.e.*, homozygous for the mutant allele) were pooled with equal representation. A small-insert library (∼300 bp) was prepared for the pooled sample and 340 million paired-end, 125 bp reads were generated by an Illumina HiSeq 2500 (BioProject: PRJNA335519). The short reads were mapped to the LF10 genome assembly version 1.8 (http://monkeyflower.uconn.edu/resources/) using CLC Genomics Workbench 7.0 (QIAGEN, Valencia, CA).

Theoretically, there should be very few single-nucleotide polymorphisms (SNPs) of near 100% frequency in the “F_2_ reads – LF10 genome” alignment, because the only differences between the mutant genome and the LF10 genome are the EMS-induced mutations. Among those mutations, the vast majority are unlinked to the causal gene and should segregate randomly in the F_2_ population with SNP frequency closer to 50% than 100%. However, in reality, there are often thousands or tens of thousands such high-frequency SNPs after short-read mapping, due to assembly error in the reference genome and nonspecific mapping of repetitive sequences. One way to filter out these artifactual high-frequency SNPs is to compare the SNP profiles of multiple bulk segregant analysis experiments, as these artifactual SNPs are expected to be shared between multiple mutant SNP profiles, whereas the true causal mutation should be unique to each mutant. Therefore, in addition to ML10422, we also mapped the short reads from four previously published mutants, *guideless* (Yuan *et al.* 2013b), *rcp1* (Sagawa *et al.* 2016), *act1-D* (Ding *et al.* 2017), and *rcp2* (Stanley *et al.* 2017), to the LF10 reference genome for between-mutant comparisons.

The 134,456 raw SNPs that resulted from the ML10422 mapping were first filtered by frequency (>95%) and depth of coverage (<200). SNPs with >200-fold coverage were discarded because these regions are highly repetitive and the reads were likely to be mapped incorrectly. The remaining 36,247 SNPs were then compared with the other four mutant profiles sequentially using a customized perl script (available upon request): 2603 SNPs remained after comparison to *guideless*, 2361 SNPs remained after comparison to *rcp1*, 33 SNPs remained after comparison to *act1-D*, and after comparison to *rcp2* only 20 SNPs unique to ML10244 were left.

### Chemical analyses of petal flavonoids

Flavonoid extraction and identification essentially followed Lin and Harnly (2007). For each genotype, petals from two flowers were ground in a glass tissue homogenizer with 60% (v/v) methanol solution for 2 min, after which time the material appeared to be completely pulverized. The solution containing the tissue was then transferred to a glass test tube and sonicated for 1 hr using a Fisher Scientific FS20 bath type sonicator. Ice was added to the bath approximately every 15 min to prevent excessive heating of the samples. Following sonication, the solution was centrifuged for 1 min at 13,600 × *g* and then filtered using 4 mm Millex HV (0.45 μm) syringe filters to remove all debris.

Absorption spectra of the sonicated extracts were measured from 200 to 600 nm in a 2 mm path cuvette using a Varian Cary 50 UV/Vis spectrometer and compared to those reported by Harborne (1984) and the *bona fide* apigenin and apigenin-7-glucuronide standards (Sigma-Aldrich).

Tandem mass spectrometry (MSMS) analyses were performed using an Applied Biosystems API2000 instrument in ESI mode with the following conditions: curtain gas, 20 psi; ionspray voltage, −4200 V; temperature, ambient; ion source gas, 20 psi; ion source gas 2, 6 psi; declustering potential, −20 V; focusing potential, −200 V; entrance potential, −10 V; scan intervals, 0.5 sec. The collision energy was −50 eV. Mobile phase was acetonitrile delivered at a rate of 50 μl/min.

High-performance liquid chromatography (HPLC) for separation of pigments was conducted using a Waters 600 HPLC system equipped with a 2996 photodiode array detector and a Waters Atlantis dC18 analytical column (4.6 × 250 mm, 5 μm). The mobile phase protocol was as described in Lin and Harnly (2007).

### Plasmid construction and plant transformation

To build the RNA interference (RNAi) construct for *FNS* knockdown, a 328 bp fragment from the second exon of *MlFNS* was amplified with Phusion High-Fidelity DNA Polymerase (New England Biolabs) using a pair of primers with added restriction sites for cloning: *MlFNS*_RNAi_F (5′-GTTCTAGACCATGGACGAAACCCCAAGATCTGGGAA-3′) and *MlFNS*_RNAi_R (5′-GTGGATCCGGCGCGCCTCAATTCCCCGAAACTACAACG-3′). The amplified fragment was inserted into the binary vector pFGC5941 (Kerschen *et al.* 2004) by a two-step procedure. First the PCR product was cloned into the original pFGC5941 vector in the sense orientation as an *Asc*I/*Nco*I fragment, and then the same PCR product was cloned in the antisense orientation into the *Bam*HI/*Xba*I site of the plasmid that already contained the sense fragment. The final plasmid construct was verified by sequencing and then transformed into *Agrobacterium tumefaciens* strain GV3101 for subsequent plant transformation (into the wild-type LF10).

Plant transformation was conducted as detailed in Yuan *et al.* (2013a). *A. tumefaciens* was cultured in 300 ml of sterilized LB broth (Fisher Scientific) containing kanamycin (50 mg/L), rifampicin (25 mg/L), and gentamicin (50 mg/L). The entire culture was pelleted and resuspended in 250 ml of 5% sucrose solution, which contained 0.1 M acetosyringone and 0.1% (v/v) of surfactant Silwet L-77 (OSi Specialties). Early-stage flower buds (<5 mm) were sprayed directly with the inoculation solution until thoroughly soaked. The entire plants were then placed into a vacuum chamber and held at 28–30 in Hg for 2 min before a rapid release of pressure. Postinfiltrated plants were maintained in plastic tents with high humidity for a 24 hr period before returning them to the greenhouse. Flowers were hand pollinated and the resulting seeds were densely sowed in 25 × 30 cm flats. Transgenic glufosinate-resistant plants were selected by daily spraying the young seedlings with 1:1000 dilution of Finale (Bayer).

### Quantitative reverse transcriptase PCR

Quantitative reverse transcriptase PCR (qRT-PCR) was performed to determine the relative expression level of *FNS* and the ABP genes (*MlCHSa*, *MlCHI*, *MlF3Ha*, *MlDFR*, and *MlANS*) in the RNAi lines compared to the wild-type. Total RNA was isolated from 10 mm corolla using the Spectrum Plant Total RNA Kit (Sigma-Aldrich). For each sample, we treated 1 μg of total RNA with amplification grade DNaseI (Invitrogen) before cDNA syntheses using the SuperScript III First-Strand Synthesis System (Invitrogen). cDNA samples were diluted 10-fold before qRT-PCR, which was performed using iQ SYBR Green Supermix (Bio-Rad) in a CFX96 Touch Real-Time PCR Detection System (Bio-Rad). Samples were amplified for 40 cycles of 95° for 15 sec and 60° for 30 sec. *MlUBC* was used as the reference gene (Yuan *et al.* 2013a). Primer amplification efficiencies were determined using critical threshold values obtained from a dilution series (1:4, 1:8, 1:16, and 1:32) of pooled cDNA. Primer sequences are listed in Supplemental Material, Table S1 in File S1. Relative expression of the target gene compared to the reference gene was calculated using the formula (*E*_ref_)^CP(ref)^/(*E*_target_)^CP(target)^.

### Data availability

Seeds are available upon request. File S1 contains all supplemental data, including four supplemental figures and three supplemental tables. The annotated *FNS* sequence is deposited in GenBank under accession number KX710102. Illumina short-read data have been deposited in the NCBI Short Read Archive (BioProject: PRJNA335519).

## Results

For each of the three mutant lines (ML10422, ML12540, and ML14138), the M_2_ generation showed a 1:2:1 segregation ratio of light pink:dark pink:very pale in flower color (Figure 1A and Table S2 in File S1). Backcrossing a very pale individual of each mutant line to LF10 produced F_1_ progenies that were all dark pink, and a relatively large ML10422 × LF10 F_2_ population (455 individuals) used for bulk segregant analysis (see *Materials and Methods*) again showed a 1:2:1 segregation ratio among the three phenotypes (112:235:98). These results suggest that the mutant phenotype is caused by a single locus and that the very pale individuals are homozygous for the mutant allele (*fns/fns*), whereas the dark pink individuals are heterozygous (*FNS/fns*). In addition, pair-wise complementation crosses between homozygous mutants (*i.e.*, very pale) from the three mutant lines all resulted in very pale offspring, indicating that the three mutants represent different alleles of the same locus.

**Figure 2:**
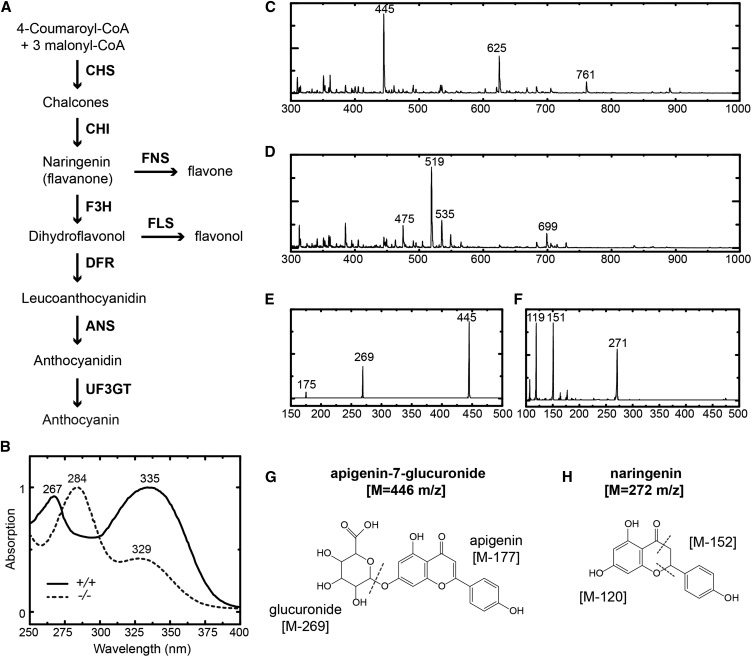
Flavonoid analyses of petal extracts. (A) A schematic of the anthocyanin biosynthetic pathway, with branches leading to flavone and flavonol biosyntheses. (B) Absorption spectra of the wild-type LF10 (solid line) and homozygous mutant (dotted line) petal extracts in 60% methanol solution. Spectra were normalized to one at the absorption maxima. The heterozygous mutant showed the same spectrum as the wild-type. (C and D) MS-ESI (negative mode) of flavonoid extract from wild-type petals (C) and homozygous mutant petals (D). (E) MSMS-ESI of the 625 m/z molecular ion in (C) (the 761 m/z molecular ion showed a very similar fragmentation pattern). (F) MSMS-ESI of the 519 m/z molecular ion in (D). (G and H) Chemical structures of apigenin-7-glucuronide (G), naringenin (H) and the respective cleavage patterns identified in (E) and (F) indicated by dotted lines. Chemical structures (G and H) were generated using ChemDrawPrime software (v16.0). ANS, anthocyanidin synthase; CHI, chalcone isomerase; CHS, chalcone synthase; DFR, dihydroflavonol 4-reductase; ESI, electrospray ionization; F3H, flavonoid 3-hydroxylase; FLS, flavonol synthase; FNS, flavone synthase; MS, mass spectrometry; UF3GT, UDP-3-O-glucosyltransferases.

The combination of bulk segregant analysis and comparison of SNP profiles between multiple EMS mutants (see *Materials and Methods*) narrowed the causal mutation of ML10422 down to 20 candidate SNPs, the vast majority of which locate in noncoding, repetitive sequences (Table S3 in File S1). Only two of the 20 candidates are nonsynonymous mutations in coding DNAs, one of which causes a drastic amino acid replacement (M371K) at a highly conserved site of the enzyme FNS (GenBank: KX710102) (Figure 1B, and Figure S1 and Figure S2 in File S1). This is interesting because FNS utilizes the same substrate, flavanone, as the anthocyanin biosynthetic enzymes (Figure 2A), and the colorless flavone products generated by FNS are structurally similar to anthocyanins and have been shown to enhance anthocyanin stability as copigments in numerous plant species [Goto and Kondo 1991; Yabuya *et al.* 2000; Kalisz *et al.* 2013; and see recent review by Trouillas *et al.* (2016)].

**Figure 3:**
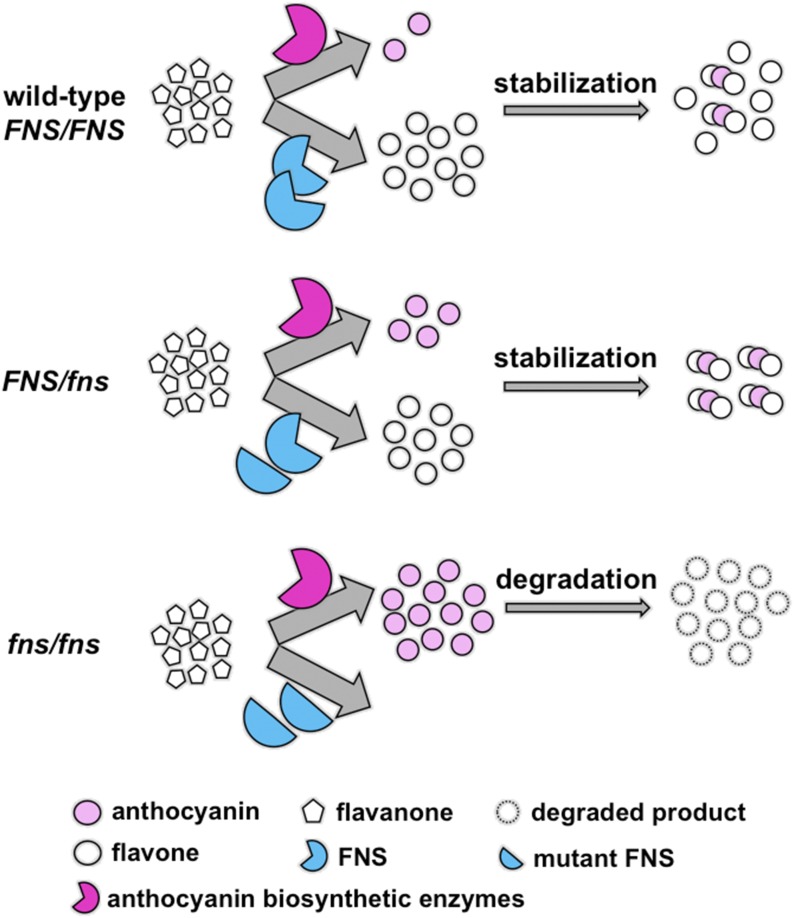
Schematic illustration of the antagonistic pleiotropic effects of the mutant *FLAVONE SYNTHASE* (*FNS*) allele in anthocyanin accumulation.

The *FNS* mutation immediately offers a potential explanation for the overdominant behavior, as illustrated in Figure 3. Assuming the drastic amino acid replacement (M371K) is a loss-of-function mutation, the heterozygote flower produces only half of functional FNS enzymes compared to the wild-type, leading to less competition with the anthocyanin biosynthetic enzymes for substrates (*i.e.*, flavanone) and consequently more anthocyanin production. Meanwhile, the half dosage of functional FNS enzymes still produces sufficient flavones to stabilize the anthocyanins. The final outcome is a dark pink flower in the heterozygote. The homozygote *fns/fns* flower produces no functional FNS enzymes and thus no competition to anthocyanin biosynthesis. However, the complete lack of flavone copigments leads to rapid degradation of anthocyanins and the final product is a very pale flower.

This model makes three clear predictions: (i) the other allelic mutants, ML12540 and ML14138, should also harbor putative loss-of-function mutations in *FNS*; (ii) the homozygous mutant should be devoid of flavones but may accumulate flavanone precursors, whereas the heterozyote and the wild-type produce plenty of flavones; and (iii) knocking down *FNS* expression in the wild-type should result in a gradient of flower color intensity with different flavone–flavanone profiles, depending on the extent of the knockdown.

Sequencing the *FNS* gene from ML12540 and ML14138 revealed a mutation in each that also causes nonconservative amino acid replacement at highly conserved sites (Figure 1B and Figure S1 in File S1), consistent with the first prediction. These multiple alleles support that *FNS* is the causal gene (more evidence from transgenic experiments are described in a later paragraph), and were renamed *fns-1* (ML10422), *fns-2* (ML12540), and *fns-3* (ML14138).

To test the second prediction, petal extracts in a 60% methanol (v/v) solution were first analyzed by UV/Vis spectroscopy. The absorption spectra revealed that the wild-type and *FNS-1/fns-1* heterozygote both exhibited two major absorption peaks at 267 and 335 nm (Figure 2B), a feature of apigenin-like flavones (Harborne 1984), whereas the *fns-1*/*fns-1* homozygote exhibited two major absorption peaks at 284 and 329 nm, a feature of glycosylated naringenin (*i.e.*, flavanone) (Harborne 1984). Anthocyanins, which display a characteristic band between 475 and 560 nm (Harborne 1984), were not observed in the spectra of any genotype due to rapid postextraction degradation.

To further characterize the molecular identities of these flavonoids, the samples were analyzed by MSMS (Figure 2 and Figure S3 in File S1). MSMS fragmentation patterns revealed that the three most prominent ions from the wild-type sample (761, 625, and 445 m/z) all display product ions at 445 and 269 m/z (Figure 2E), which are consistent with apigenin-7-glucuronide and free apigenin, respectively, a pattern confirmed by fragmentation of a *bona fide* apigenin-7-glucuronide standard (Figure S3, A–D in File S1). In contrast, the prominent ions from the homozygous mutant sample (Figure 2D) are consistent with glycosylated derivatives of the flavanone naringenin (*e.g.*, 699, 519, and 475 m/z, Figure 2H), and its hydroxylated form eriodictyol (535 m/z, Figure S3, E and F in File S1). Fragmentation of the 519 m/z molecular ion reveals masses at 272 (free naringenin), 151, and 119 m/z (Figure 2F). The latter two fragments are consistent with the cleavage of naringenin in a pattern reported for similar flavonoid structures by Lin and Harnly (2007) and Wojakowska *et al.* (2012), wherein cleavage occurs between C3–C4 and O1–C2 (Figure 2H). Similarly, fragmentation of the 535 m/z ion reveals a mass at 287 m/z (free eriodictyol), with subsequent cleavage of the C3–C4 and O1–C2 bonds to form the 151 and 119 m/z fragments (Figure S4E in File S1).

**Figure 4:**
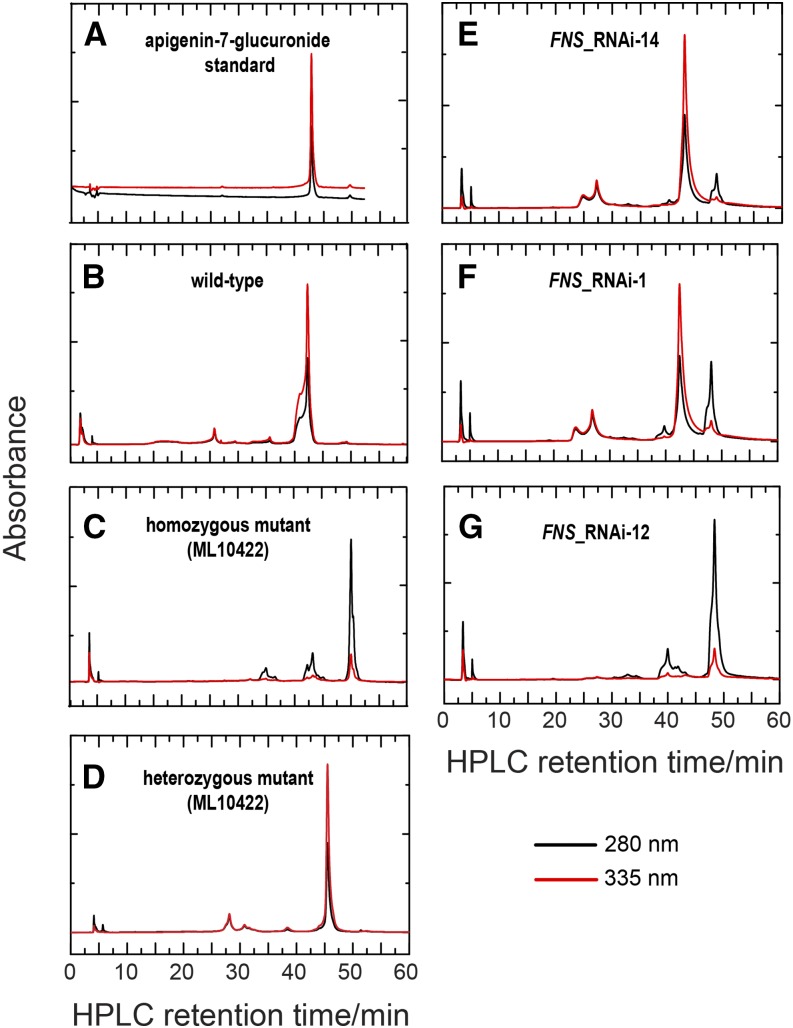
HPLC analyses. Shown are the HPLC chromatograms of the apigenin-7-glucuronide standard (A) and whole petal extracts from the wild-type LF10 (B), the homozygous (C) and heterozygous (D) mutants, and three representative RNAi lines (E–G). Red and black lines represent detection at wavelengths 335 and 280 nm, the absorption maxima that distinguish flavones and flavanones, respectively. The flavone eluted at 43 min, and flavanone at 50 min. Note that a variation of 1–2 min between runs in the retention times of the same eluting pigments is not uncommon for this HPLC protocol. HPLC, high-performance liquid chromatography; RNAi, RNA interference.

Cochromatography with *bona fide* standards conducted as part of the HPLC analyses also indicates that apigenin-7-glucuronide is the primary flavone component in petal extracts of the wild-type as well as the *FNS-1*/*fns-1* heterozygote, but is completely absent from the homozygous mutant petal, where the flavanone precursors accumulate (Figure 4). The presence of flavanone precursors in the homozygous mutant, in conjunction with the absence of the corresponding flavones, provides strong evidence that the *in planta* function of the identified FNS protein is indeed to convert flavanone to flavone, consistent with sequence analysis (Figure S2 in File S1) and confirming the second prediction.

**Figure 5:**
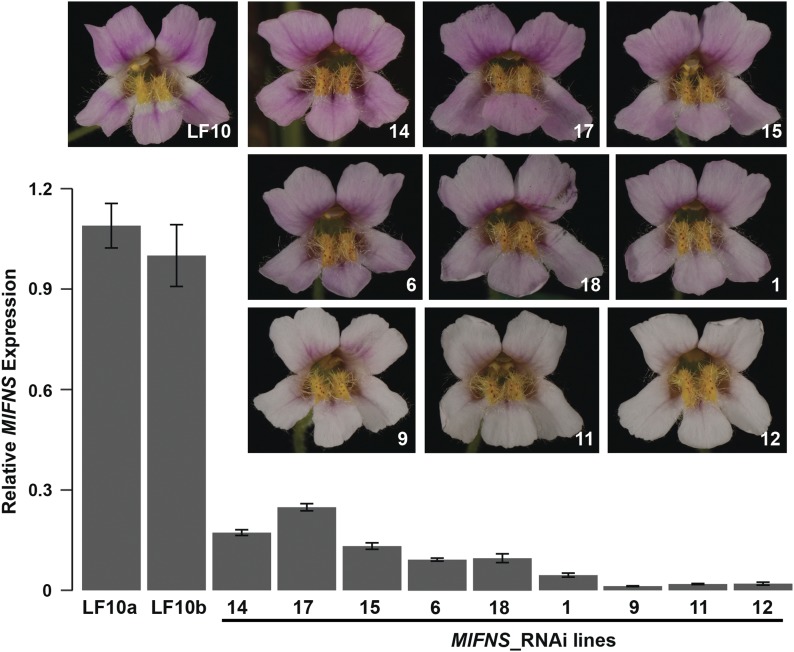
Downregulation of *FNS* using RNAi in the wild-type background generates a gradient of flower color intensity. Flower images of nine representative RNAi lines are arranged according to their color intensity, which is inversely correlated with the extent of RNA knockdown. Relative transcript level of *FNS* in 10 mm corolla was measured by qRT-PCR. *MlUBC* was used as the reference gene. Error bars represent 1 SD from three technical replicates. qRT-PCR, quantitative reverse transcriptase polymerase chain reaction; RNAi, RNA interference.

To test the third prediction, we constructed an RNAi plasmid with a 328 bp fragment from the second exon of *FNS* and transformed it into the wild-type background. This 328 bp fragment was chosen to minimize off-target effects; no other genomic regions perfectly match this fragment for a contiguous block longer than 16 bp, as determined by BLASTing against the LF10 genome assembly with an *E*-value cutoff of 0.1. We obtained 25 independent stable RNAi transgenic lines displaying a gradient of flower color intensity (Figure 5). qRT-PCR at the 10 mm corolla developmental stage showed a general inverse relationship between color intensity and the extent of *FNS* knockdown: ∼80% knockdown corresponds to color intensity at or above the wild-type level (*e.g.*, lines 14 and 17); >95% knockdown corresponds to the very pale phenotype (*e.g.*, lines 9, 11, and 12); and ∼90% knockdown leads to intermediate phenotypes (*e.g.*, lines 1 and 6) (Figure 5). Furthermore, qRT-PCR of the anthocyanin biosynthetic genes showed no significant downregulation in the three very pale RNAi lines (lines 9, 11, and 12) compared to the wild-type (Figure S4 in File S1), indicating that *FNS* knockdown is solely responsible for the very pale phenotype.

HPLC analyses showed that petal extract of a weak knockdown line (line 14) contains largely flavones, but also accumulates a small proportion of flavanone precursors (Figure 4E), whereas a severe line (line 12) completely lacks flavones and only accumulates flavanones (Figure 4G), resembling the homozygous mutant; an intermediate line (line 1) has an intermediate flavone–flavanone profile (Figure 4F). These results confirmed the third prediction. The fact that transgenic lines with severe *FNS* knockdown could fully recapitulate the homozygous *fns/fns* mutant phenotype, in both flower color appearance and chemical composition, suggests that the three independent mutant alleles are indeed all loss-of-function alleles and provides further evidence that the *in vivo* function of the *FNS* product identified herein is responsible for the conversion of flavanone to flavone.

## Discussion

Three lines of evidence (*i.e.*, multiple mutant alleles, chemical composition of petal extracts, and RNAi phenotypes) support our model explaining the flower color overdominance. On one hand, the loss-of-function *fns* alleles positively affect one component of the phenotypic output (*i.e.*, anthocyanin production) by alleviating competition with the anthocyanin biosynthetic enzymes. On the other hand, they negatively affect another component of the phenotypic output (*i.e.*, anthocyanin maintenance) by compromising the stabilization capacity. An overdominant phenotype could be potentially achieved by maximizing the positive effects and meanwhile minimizing the negative effects of the mutant allele (*e.g.*, in a heterozygous state, see Figure 3).

The maintenance of anthocyanins by stabilizing copigments has been reported in many systems and has been modeled computationally [reviewed by Trouillas *et al.* (2016)]. When the production of anthocyanins and the copigments (*e.g.*, flavones) requires the same substrate, the enzyme located at the branching point of these biochemical pathways (*e.g.*, anthocyanin *vs.* flavone biosynthesis) plays a critical role in balancing the metabolic flux and the ultimate phenotypic output. This model provides insights into the challenges that underlie anthocyanin biofortification in crops and ornamental plants, an area of great interest due to the reported benefits of these antioxidant compounds to human health (Gould *et al.* 2008) and their significant value to the floriculture industry (Passeri *et al.* 2016). A notable example is that transgenic suppression of *FNS* in an attempt to increase anthocyanin concentration in *Torenia* flowers resulted in an unexpected decrease (Ueyama *et al.* 2002), which was interpreted as a consequence of destabilization of anthocyanins due to the lack of flavones. However, an overdominant effect was not detected in the *Torenia* study, probably because the *FNS* knockdown was too severe, just as in our very pale RNAi lines.

It is worth noting that the *M. lewisii* overdominant color in the *FNS/fns* heterozygote is by no means a peculiar example of single-gene overdominance. A study of mutant recessive alleles in maize revealed several loci showing single-gene overdominance for total yield (Dollinger 1985). With no exception, the mutant allele at every locus produced antagonistic pleiotropic effects, positively affecting some components and negatively affecting other components of yield. Although the molecular nature of these maize alleles is unknown, these observations support antagonistic pleiotropy (*i.e.*, alleles with opposing effects on different components of the phenotypic output) as a potential general mechanism of single-locus overdominance.

In fact, molecular evidence of the few well-known examples of single-gene overdominance in plants further supports this view. An early study on alcohol dehydrogenase (Adh) activity in maize (Schwartz and Laughner 1969) revealed a molecular basis for single-gene overdominance that is similar to our results. The Adh enzyme is a dimer. The autodimer in one inbred line has high activity but low stability, whereas the autodimer in another inbred line has low activity but high stability. The allodimer formed in the heterozygote is both active and stable, a clear case of single-gene overdominance caused by antagonistic pleiotropy. Another example is the loss-of-function *single flower truss* (*sft*) allele in tomato (Krieger *et al.* 2010). Total fruit yield is <50% of the wild-type in the homozygous condition, but >160% of the wild-type in the heterozygous condition. Compared to the wild-type allele, the *sft* allele negatively affects fruit yield by delaying flowering and reducing flower number per inflorescence, but positively affects fruit yield by altering tomato growth habit and producing more inflorescences per plant, achieving the maximum phenotypic output in the heterozygous condition.

Given that virtually all phenotypic traits can be decomposed into multiple components (*e.g.*, rice yield can be decomposed to the number of grains and the average weight per grain), in conjunction with the vast knowledge base of gene function accumulated in the past few decades, the principle of antagonistic pleiotropy could be readily implemented to guide searching for overdominant alleles in available germplasm or engineering such alleles through genomic editing for crop improvement.

## Supplementary Material

Supplemental material is available online at www.g3journal.org/lookup/suppl/doi:10.1534/g3.117.300336/-/DC1.
