# Supplementary material for: Molecular Basis of Overdominance at a Flower Color Locus
Source: G3 (Bethesda). 2017 Oct 19;7(12):3947–54. doi: 10.1534/g3.117.300336 (PMC5714491; doi:10.1534/g3.117.300336)
Supplement: Supplementary file 1 [file 3947FileS1.pdf]

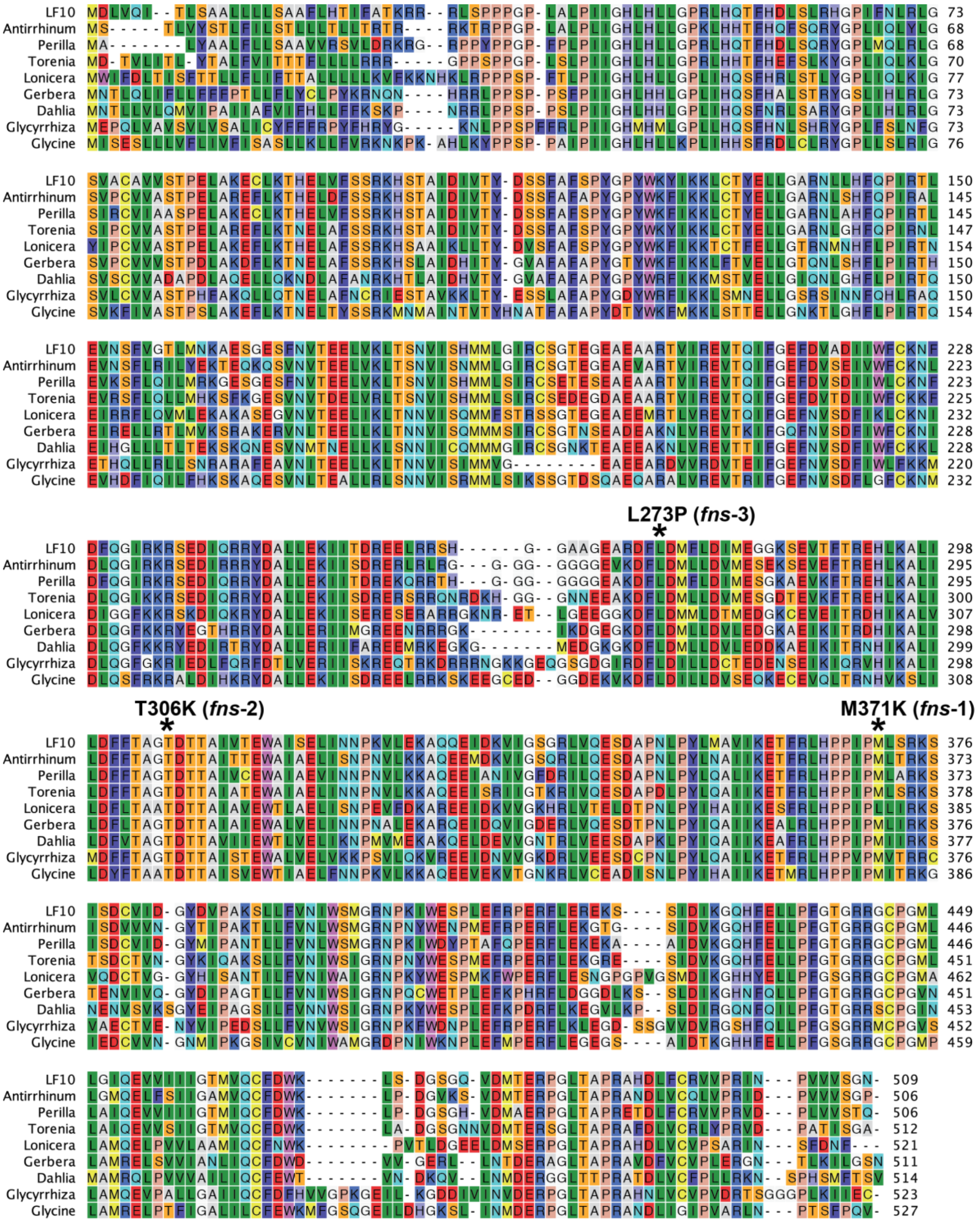

Figure S1. Alignment of *Mimulus lewisii* FNS and its orthologs from other plant species. Sequences other than *M. lewisii* FNS were retrieved from GenBank (with accession numbers shown in Figure S2 below). Asterisks indicate non-conservative amino acid replacements at highly conserved sites.

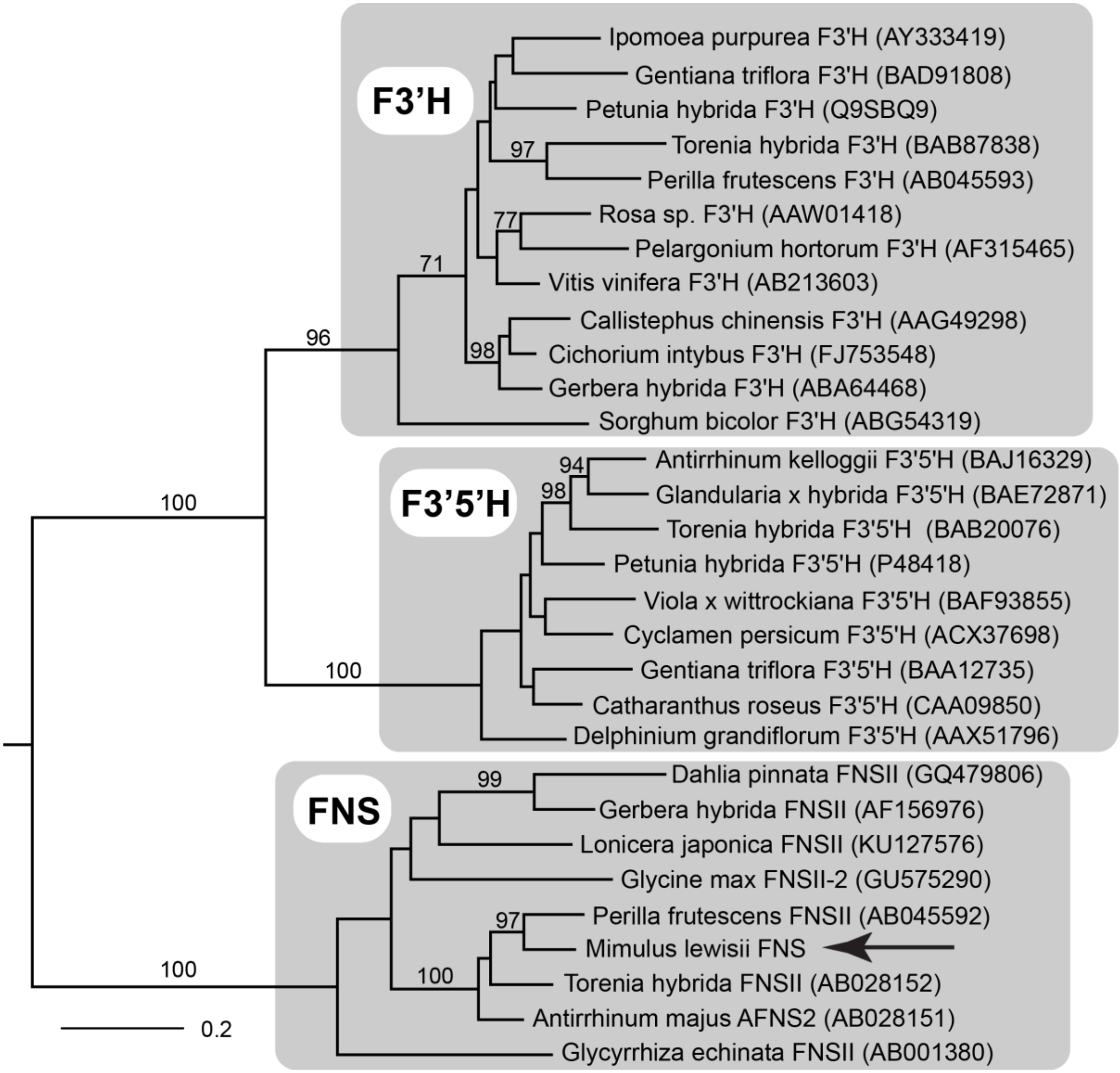

Figure S2. A maximum likelihood (ML) phylogeny inferred from the amino acid sequences of the three types of P450 enzymes involved in flavonoid biosynthesis. FNS: Flavone synthase; F3'H: Flavonoid 3'-hydroxylase; F3'5'H: Flavonoid 3',5'-hydroxylase. The arrow indicates that the causal gene sequence is clearly clustered with other known flavone synthases. All sequences other than *M. lewisii* FNS were retrieved from GenBank (accession numbers are shown on the right of the species name). ML analysis was conducted using the RAxML web-server (http://embnet.vital-it.ch/raxml-bb/), with the JTT amino acid substitution matrix and the GAMMA model of rate heterogeneity. Clade support was estimated by 100 bootstrap replicates. Bootstrap values >50% are indicated along the branches. The tree is rooted by midpoint rooting.

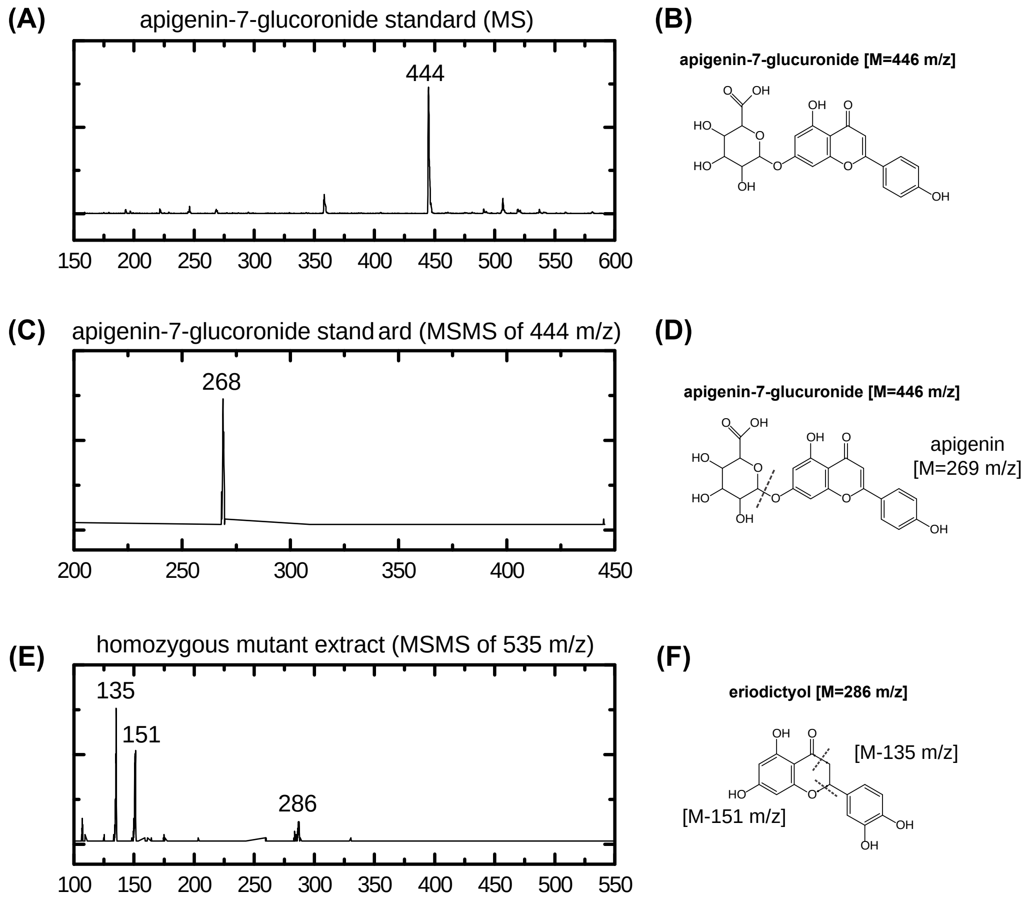

Figure S3. Flavonoid analyses. (A) MS-ESI (negative mode) of the apigenin-7-glucoronide standard and (B) corresponding chemical structure. (C) MSMS-ESI of the 444 m/z molecular ion in (A), and (D) the chemical structure of apigenin-7-glucuronide with cleavage pattern indicated by the dotted line. (E) MSMS-ESI (negative mode) of 535 m/z ion shown in Figure 2d, and the chemical structure of eriodictyol with cleavage pattern indicated by dotted lines. Chemical structures in (B,D,F) were generated using ChemDrawPrime software (v16.0).

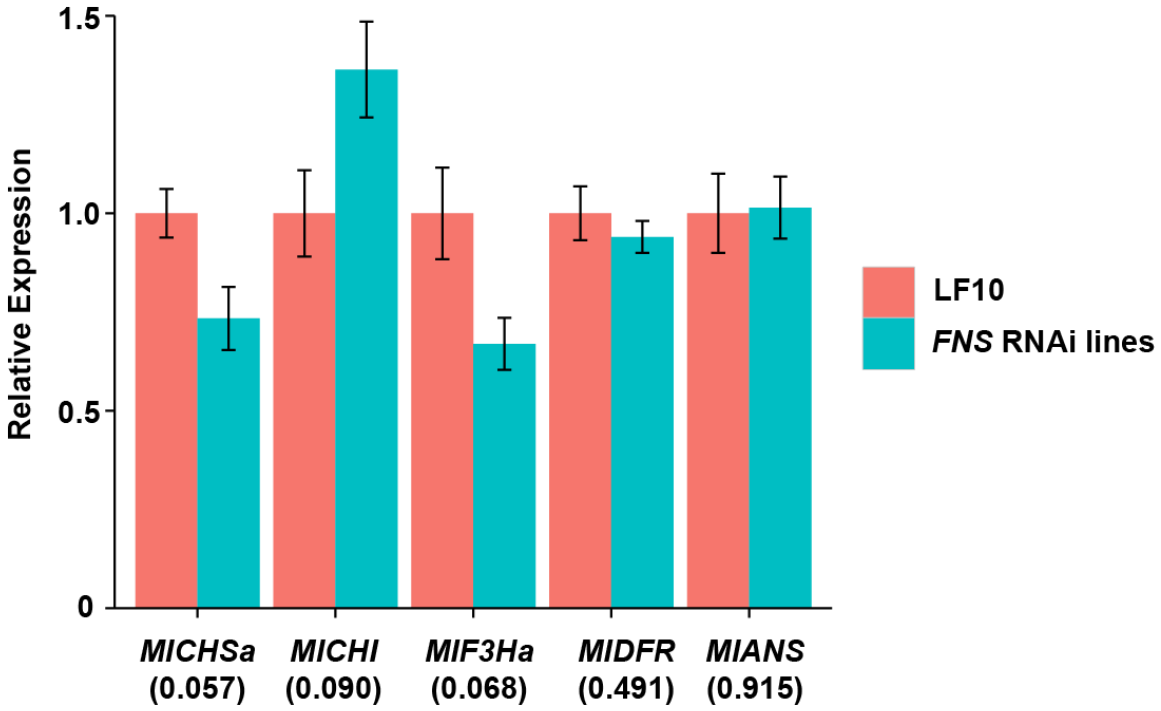

Figure S4. Relative transcript levels of anthocyanin biosynthetic genes in severe *FNS* RNAi lines compared to the wild-type LF10 (10-mm corolla), as measured by qRT-PCR. *MlUBC* was used as the reference gene. Error bars represent 1 SD from three biological replicates. The *FNS* RNAi replicates were represented by three lines (line-9, 11, and 12); the wild-type replicates were represented by three LF10 plants. P-values calculated by two-tailed t-test are given in parentheses below the name of each gene.

Table S1. RT-PCR primers used in this study.

PRIMER	FORWARD (5'-3')	REVERSE (5'-3')
MlUBC	GGCTTGGACTCTGCAGTCTGT	TCTTCGGCATGGCAGCAAGTC
MlFNS	GAGGGTGGCAAATCGGAGGTGA	CGTCTCCTTGATGACCGCCATA
MlCHSa	GCACCGTCCTCCGCATGGCCAAG	TGCCCGACGAGACTGTCCAAATG
MlCHI	AAACTTCCCGCCTGGGAGCTCCA	TACACCATGCCTCCCAATGATCGA
MlF3Ha	AACCCGATCTCACACTAGGCGTC	CTTCAACGGGTAAACTATGGCGTT
MlDFR	TCGAGGATCCCACAGCACAAGGA	TGGCTTCTCTAAACATGTCCTCCA
MlANS	CCCAAGATTCCGGCTGATTACGT	TCGTGTTCTAATCCTAGTCCGGT

Table S2. Segregation ratio for mutant lines.

MUTANT POPULATION	NUMBER OF PLANTS (LIGHT PINK: DARK PINK: VERY PALE)	*P* FOR 1:2:1 (χ^2, *df* =2)
ML10422 M_2	6:15:7	0.898 (χ^2= 0.214)
ML12540 M_2	7:10:4	0.636 (χ^2= 0.905)
ML14138 M_2	4:9:5	0.946 (χ^2= 0.111)
ML10422 x LF10 F_2	112:235:98	0.319 (χ^2= 2.285)

Table S3. Candidate SNPs from the mutant genome comparisons. The SNP highlighted in bold is the causal mutation.

LF10g_v1.8 SCAFFOLDS	POSITION	WILD-TYPE	MUT-ANT	ANNOTATION
scaffold40	125711	G	A	Non-coding, repetitive sequence
scaffold342	402726	-	T	Non-coding sequence
scaffold575	292986	C	T	Synonymous substitution in a Reticulon gene
scaffold586	4144	G	A	Non-coding, repetitive sequence
scaffold621	92999	T	A	Mutator-like transposon
**scaffold858**	**116982**	**T**	**A**	**Non- Synonymous substitution in a Cytochrome P450 Flavone synthase II gene**
scaffold877	571866	G	C	Non-coding sequence
scaffold1010	97910	C	T	LTR-retrotransposon
scaffold1956	21075	C	T	Non-coding sequence
scaffold1987	6849	A	C	Non-synonymous substitution in a Trehalose-phosphatase gene
scaffold2014	37525	G	T	Non-coding, repetitive sequence
scaffold2086	22242	A	G	Non-coding sequence
scaffold2510	12091	C	A	Non-coding, repetitive sequence
scaffold2510	12178	A	T	Non-coding, repetitive sequence
scaffold2528	30135	C	A	Mutator-like transposon
scaffold2873	1448	T	C	Non-coding, repetitive sequence
scaffold3328	3747	C	T	Helitron-like transposon
scaffold4959	72849	G	T	Non-coding, repetitive sequence
scaffold4995	233	C	A	Non-coding, repetitive sequence
scaffold3047	13507	G	T	Non-coding, repetitive sequence
